# Early Life Origins of Lung Ageing: Early Life Exposures and Lung Function Decline in Adulthood in Two European Cohorts Aged 28-73 Years

**DOI:** 10.1371/journal.pone.0145127

**Published:** 2016-01-26

**Authors:** Julia Dratva, Elisabeth Zemp, Shyamali C. Dharmage, Simone Accordini, Luc Burdet, Thorarinn Gislason, Joachim Heinrich, Christer Janson, Deborah Jarvis, Roberto de Marco, Dan Norbäck, Marco Pons, Francisco Gómez Real, Jordi Sunyer, Simona Villani, Nicole Probst-Hensch, Cecilie Svanes

**Affiliations:** 1 Swiss Tropical and Public Health Institute, Dept. Epidemiology and Public Health, Basel, Switzerland; 2 University of Basel, Basel, Switzerland; 3 Allergy and Lung Health Unit, Centre for Epidemiology and Biostatistics, School of Population and Global Health, The University of Melbourne, Melbourne, Australia; 4 Unit of Epidemiology and Medical Statistics, Department of Public Health and Community Medicine, University of Verona, Verona, Italy; 5 Hôpital Intercantonal de la Broye, Payerne, Switzerland; 6 Dept. of Respiratory Medicine and Sleep, Landspitali University Hospital Reykjavik, Reykjavik, Iceland; 7 Helmholtz Center Munich, National Research Centre for Environmental Health, Munich, Germany; 8 Ludwig Maximilians University Munich, University Hospital Munich, Institute and Outpatient Clinic for Occupational, Social and Environmental Medicine and German Center for Lung Research (DZL), Munich, Germany; 9 Department of Medical Sciences: Respiratory Medicine & Allergology, Uppsala University, Uppsala, Sweden; 10 Department of Public Health Sciences, Imperial College London, London, United Kingdom; 11 Department of Medical Sciences: Occupational & Environmental Medicine, Uppsala University, Uppsala, Sweden; 12 Division of Pulmonary Medicine, Regional Hospital of Lugano, Lugano, Switzerland; 13 Department of Clinical Science, University of Bergen, Bergen, Norway; 14 Department of Obstetrics and Gynaecology, Haukeland University Hospital, Bergen, Norway; 15 Centre for Research in Environmental Epidemiology (CREAL), Barcelona, Spain; 16 University of Pavia, Faculty of Medicine, Dept. of Public Health, Neurosciences, Experimental and Legal Medicine, Pavia, Italy; 17 Bergen Respiratory Research Group, Centre for International Health, University of Bergen, Bergen, Norway; 18 Department of Occupational Medicine, Haukeland University Hospital, Bergen, Norway; University Children's Hospital Basel, SWITZERLAND

## Abstract

**Objectives:**

Early life environment is essential for lung growth and maximally attained lung function. Whether early life exposures impact on lung function decline in adulthood, an indicator of lung ageing, has scarcely been studied.

**Methods:**

Spirometry data from two time points (follow-up time 9–11 years) and information on early life exposures, health and life-style were available from 12862 persons aged 28–73 years participating in the European population-based cohorts SAPALDIA (n = 5705) and ECRHS (n = 7157). The associations of early life exposures with lung function (FEV_1_) decline were analysed using mixed-effects linear regression.

**Results:**

Early life exposures were significantly associated with FEV_1_ decline, with estimates almost as large as personal smoking. FEV_1_ declined *more rapidly* among subjects born during the winter season (adjusted difference in FEV_1_/year of follow-up [95%CI] -2.04ml [-3.29;-0.80]), of older mothers, (-1.82 ml [-3.14;-0.49]) of smoking mothers (-1.82ml [-3.30;-0.34] or with younger siblings (-2.61ml [-3.85;-1.38]). *Less rapid* FEV_1_-decline was found in subjects who had attended daycare (3.98ml [2.78;5.18]), and indicated in subjects with pets in childhood (0.97ml [-0.16;2.09]). High maternal age and maternal smoking appeared to potentiate effects of personal smoking. The effects were independent of asthma at any age.

**Conclusion:**

Early life factors predicted lung function decline decades later, suggesting that some mechanisms related lung ageing may be established early in life. Early life programming of susceptibility to adult insults could be a possible pathway that should be explored further.

## Introduction

Early childhood is a critical time window for subsequent lung health. Adverse childhood environmental exposures can restrain growth[1], modulate lung function [1, 2] and induce changes to gene-expression, modulating airway pathophysiology.[3, 4] The impact of a range of early life factors have been evidenced such as parental life-style [5, 6], nutrition [7] ambient air pollution [8, 9] or viral infections.[10] Epigenetic programming has been suggested as an underlying mechanism leading to less favourable long term respiratory health outcomes. [7, 11–13]

Emerging research suggests that not only lung growth, but also lung ageing, may be programmed early in life [14, 15]. Lung ageing encompasses the physiological as well as pathological processes which lead to altered lung function and lung diseases with increasing age. [16] Lung function decline is on one hand a normal ageing process, on the other hand it can be potentiated by risk factors, such as smoking [17, 18] or obesity.[17, 19] Early life impact on adult respiratory health is relatively well documented [20–23]. However, the potential impact on lung function decline by early life factors has been scarcely studied and remains inconsistent. Svanes et al., for example, shows that a childhood disadvantage score, including childhood asthma, predicted more rapid lung function decline in middle aged adults [21] and Jackson et al. evidenced earlier and quicker lung function decline for young adults with lower childhood SES [24] whereas Marossy et al. found no significant association between lung function decline in adults age 35–45 and early life respiratory infections.[25]

Smoking is known as the main adult risk factor for accelerated lung function decline; however, causes for varying susceptibility to tobacco exposure between individuals are not well understood. Early life factors, in particular parental smoking, have been hypothesized to play a role for modifying susceptibility.[26–28]. No previous study on early life factors has yet followed participants into old age or had the power to investigate individual early life factors in sufficient detail. We wished to investigate the hypotheses i) that early life factors may predict lung function decline, independent of childhood or parental asthma and ii) that one potential pathway might be through altered susceptibility to adult insults like smoking.

The combined data of the two cohorts, the Swiss Study on Air Pollution And Lung and Heart Disease In Adults (SAPALDIA) and the European Community Respiratory Health Study (ECRHS) offered the opportunity, the necessary population size and age range to study these hypotheses.

## Material and Methods

### Study population

The study population consists of 12862 subjects of the SAPALDIA [29] and the ECRHS cohort. [30] Both studies have been described in detail elsewhere. In short, the SAPALDIA study population was recruited in 1991 as a population–based, random sample of adults (N = 9651, age 20–60 years) from eight study areas in Switzerland.[29] In the second assessment in 2002/03, lung function measures as well as the questionnaire on socio-demographic characteristics, life-style factors, living, housing and work related characteristics and health status, were repeated. Lung function data from both surveys was available for 5705 participants and mean follow-up time was 10.9 years. In ECRHS I, a random sample of adults aged 20–44 years were recruited in 29 centres (N = 13 359).[30] 7157 participants with lung function measures at two time points (at ECRHS I in1991–1993 and at ECRHS II in 1998–2002), were included into the analyses. The mean follow-up time was 8.8 years. The two cohorts have coordinated study design, questionnaires and common standards for interview and clinical examinations which allows for analyses of the combined data sets. Each study centre received approval by the institutional or regional ethics committee (S1 Text), and all participants signed informed consents.

Because of the large number of study centres, and the large number of study participants from Switzerland, the study centres were categorised into four European regions: Southern (Spain, France-Montpellier, Central (Switzerland, France-Grenoble and Italy-Pavia, -Turin, -Verona), Northern (Iceland, Sweden, Norway, Estonia) and Western Europe (Great-Britain, Belgium, France-Bordeaux and -Paris; S1 Fig).

### Early life factors

Information on early life characteristics was collected by an interview-led questionnaire assessing serious respiratory infection <2 years, day care attendance <5 years, bedroom sharing, older and younger siblings, maternal age at delivery, season of birth (winter, defined as born in November to end of January, versus other seasons), paternal and/or maternal smoking during childhood, childhood pet keeping and urban living environment (large town vs. small town or farm/village) (S5 Table).

### Lung function measurements

Spirometry testing in both studies was performed according to the ECRHS protocol following the American Thoracic Society guidelines.[31] The maximum FEV_1_ and maximum forced vital capacity (FVC) of up to five technically acceptable manoeuvres were determined. Annual decline in FEV_1_ (ΔFEV_1_/yr.) was calculated by subtracting the baseline from the follow-up value and dividing the difference by the individual time of follow-up in years, a negative value representing a decline. SAPALDIA used identical spirometry devices (model 2200, SensorMedics Corp., Yorba Linda, CA, USA) and protocols in both examinations,[32] as did ECRHS centres using same or comparable spirometers (Spiro Medics; Biomedin).[21]

### Covariates

Asthma status: Participants reported doctor diagnosed asthma and age of onset. Childhood and teenage asthma was defined based on the age of reported first asthma attack (<10 yrs. of age, respectively <20 yrs.). Adult asthma was defined as having the first asthma attack after the age of 20 yrs.

Other: Socio-demographic, individual, lifestyle factors associated with respiratory health and family predisposition, such as parental and sibling asthma, were considered as confounders. Smoking status was defined on self-reported smoking history. Pack years at the 2^nd^ survey were calculated based on an estimation of the average number of cigarettes smoked per year. Missing data for pack years (N = 225) and maternal age at birth (N = 237) were imputed by a “simple” imputation technique based on multivariate regression models to predict the missing information. Imputed data were only used as confounders in multivariate analyses.

### Statistical methods

Descriptive analyses, including comparative lung function (FEV_1_) at first survey and lung function decline (ΔFEV_1_/yr.), were ran by European region and by smoking status. We studied the impact of early life factors on lung function decline in a mixed-effects linear regression model; in a first step, for single early life factors (S1 Table), and secondly, adjusting mutually for all early life exposures. Model adjustments were made for mid age, mid age square, mid BMI (mid = [survey 1 + survey 2]/2), change in BMI (between survey 1 and 2), height, pack years smoked, age at highest education, based on the significance level of p≤0.2. Different regional adjustments, including the four European regions (main analyses), the cohorts (ECRHS, SAPALDIA) or study centre as random factors, were performed. To investigate independency of the investigated early life factors from childhood & teenage and adult asthma, we ran additional models adjusting for the reported asthma status, as well as sensitivity analyses excluding subjects who reported 1.) asthma as a child (<10 yrs.) 2.) ever having had asthma, 3.) COPD, defined as pre-bronchodilator FEV_1_/FVC <0.70 [33]. The potentially increased susceptibility to adult smoking was investigated by interaction terms between significant adverse early life factors and participants’ current smoking exposure (e.g. smoking*maternal smoking), and by stratified analyses by smoking status and sex. Significance of interaction was assumed at a p-value of ≥0.1. Furthermore, we ran a sensitivity analysis excluding participants under 25 yrs. at the first survey and stratified analyses by gender, smoking status and European region. To take regional differences in population size into account we performed meta-analyses by European region. All analyses were conducted using STATA 12 (www.stata.com/stata12). The final significance level was p = 0.05 for all results.

## Results

Characteristics of the 12862 study participants by European region are provided in table 1. The early life factors, except season of birth (p = 0.7), differed significantly between the regions (p<0.001, Table 1). Age-and height-adjusted lung function and lung function decline is presented in table 2. In both men and women FEV_1_ decline was higher in the Northern European region and among current smokers (Table 2).

**Table 1:** Characteristics of the study population of the ECRHS and SAPALDIA cohorts, by European region.

	European Regions								All	
	Southern		Central		Northern		Western			
	N = 1517		N = 6530		N = 3089		N = 1726		N = 12862	
**Socio-demographic factors**†	Mean	s.d.	Mean	s.d.	Mean	s.d.	Mean	s.d.	Mean	s.d.
Age, 2^nd^ survey (yrs.)	42	7	51	11	43	7	44	7	47	10
BMI, 2^nd^ survey (kg/m^2^)	27	5	26	4	26	4	26	5	26	4
Age at highest (yrs.) educational degree	19	5	20	4	23	6	19	4	20	5
Pack years, 2^nd^ survey										
in all	15	20	11	19	9	14	10	17	11	18
in current smokers	25	20	23	21	19	16	24	20	22	20
**Early life factors**†	no.	%	no.	%	no.	%	no.	%	no.	%
Season of birth (winter)	395	26	1624	25	777	25	422	24	3218	25
Maternal age (>31 yrs.)	546	36	2023	31	871	28	474	27	3914	30
Maternal smoking	60	4	833	13	982	32	511	30	2386	19
Paternal smoking	1032	68	3716	57	1835	59	1158	67	7741	60
Severe respiratory infection	124	8	560	9	364	12	156	9	1204	9
Urban living environment	488	32	1972	30	1550	50	694	40	4704	37
Sharing bedroom	784	52	1728	27	917	30	950	55	4379	34
Daycare attendance	730	48	3029	46	1422	46	692	40	5873	46
Family pet (< 5 yrs.)	916	60	2880	44	1134	37	592	34	5522	43
Older siblings	993	65.5	3942	61	1747	57	1040	60	7722	60
Younger siblings	987	65	4012	62	1769	57	1035	60	7803	61
**Asthma**†										
Childhood asthma	49	3	190	3	155	5	107	6	501	4
Adult asthma	116	8	213	3	258	9	150	10	737	6
Paternal asthma	117	8	409	6	181	6	88	5	795	6
Maternal asthma	93	6	310	5	235	8	107	6	745	6
Sibling asthma	199	13	760	12	263	9	186	11	1408	11

^†^ Differences across European regions tested by ANOVA or Chi2 as appropriate, all p<0.001 with exception of season of birth (p-value = 0.7)

**Table 2:** Age and height adjusted FEV_1_ and FEV_1_ decline per follow-up year (ΔFEV_1_/yr.) in women and men, by European region and by smoking status.

	Adjusted FEV_1_† (ml)	s.e.	Adjusted FEV_1_ decline/year‡ (ml)	s.e.
**Women**				
Southern European Region (N = 750)	2886	15	-25.2	-1.1
Central European Region (N = 3407)	2848	7	-27.4	-0.6
Northern European Region (N = 1578)	2871	10	-30.5	-0.8
Western European Region (N = 914)	2856	13	-26.9	-1.0
Never smoker (N = 4959) §	2874	6	-26.9	-0.4
Current smoker (N = 1690) §	2816	11	-30.4	-0.7
**Men**				
Southern European Region (N = 767)	3886	22	-34.2	-1.4
Central European Region (N = 3123)	3876	11	-34.7	-0.8
Northern European Region (N = 1511)	3872	15	-39.6	-1.1
Western European Region (N = 812)	3867	20	-35.8	-1.3
Never smoker (N = 4341) §	3916	9	-34.9	-0.5
Current smoker (N = 1872) §	3781	14	-38.3	-0.8

s.e. standard error; † age and height adjusted FEV1 at 2nd survey;

^‡^ age and height adjusted difference in FEV1 (ml) per year of follow up;

^§^ additionally adjusted for European regions (fixed effect)

The associations of early life factors with adult FEV_1_ decline, analysed with mixed-effects linear regression analyses and mutual adjustment for all the early life factors, are presented in Table 3. The lung function decline was significantly more rapid in participants who were born during the winter season, to mothers >31 years at delivery, with ≥2 younger siblings or exposed to maternal smoking. Significantly less rapid lung function decline was observed in persons who had attended day care in childhood and was indicated for early life pet exposure (Table 3).

**Table 3:** The associations of early life factors with lung function decline (mutually adjusted models).

Early life factors		Adjusted difference inFEV_1_ decline †			
		in ml ‡		95% CI	p-value
Season of birth	(winter vs. other)	-2.04	-3.29	-0.80	0.001
Maternal age	(>31 vs <31 yrs.)	-1.82	-3.14	-0.49	0.007
Maternal smoking	(yes vs. no)	-1.82	-3.30	-0.34	0.016
Paternal smoking	(yes vs. no)	0.56	-0.57	1.69	0.332
Severe respiratory infection	(yes vs. no)	-0.57	-2.42	1.29	0.549
Urban living environment	(urban vs. rural)	0.62	-0.84	2.08	0.408
Daycare attendance	(yes vs. no)	3.98	2.78	5.18	0.000
Sharing bedroom	(yes vs. no)	-0.42	-1.57	0.74	0.481
Family pet (<5 years)	(yes vs. no)	0.97	-0.16	2.09	0.091
Older siblings ≥2	(≥2 vs. <2)	0.56	-1.00	2.12	0.479
Younger siblings <2	(<2 vs. ≥2)	-2.61	-3.85	-1.38	0.000

^†^ Change in FEV_1_ (ml) by follow up year—a negative coefficient implies more rapid FEV_1_ decline and a positive coefficient implies less rapid decline.

^‡^ Estimates from mixed-effects linear regression, mutually adjusted for all other early life factors investigated, and for sex, mid age, mid age square, mid BMI, change in BMI (between survey 1 and 2), height, pack years smoked, age at highest education, European region (random effect)

CI = Confidence Interval

Sensitivity analyses excluding asthmatic participants (S4 Table) and adjusting for reported asthma at any age (S5 Table) showed consistent results.

Analyses stratified by sex yielded consistent results in men and women, with the exception of parental smoking and respiratory infections (S2 Table, S2 Fig): Differential effects were observed for maternal smoking by sex, with higher effect estimates in men (ΔFEV_1_/yr. -2.23 ml [-5.11 to -0.33]) than in women (-0.57 ml [-2.34 to 1.20], p_heterogeity_ = 0.08). In non-smokers, respiratory infections were more strongly associated with FEV_1_ decline in men (p_heterogeity_ = 0.053). Separate analysis for synergistic effects between adverse early life factors and adult smoking showed that maternal smoking and higher maternal age were more strongly associated with FEV_1_ decline among current smokers than among never-smokers (S3 Table). In participants exposed to both early life factors we observed a significantly larger effect estimates compared to a single exposure (Table 4). The synergistic effects were larger in men than in women (higher maternal age & smoking -4.45 ml [-7.04 to -1.85] vs. (-3.98 ml [-6.80 to -1.14]; maternal smoking& smoking -5.87 [-10.01 to -1.7] vs. -3.17 [-6.39 to -0.08]). Further stratified analyses by sex yielded a significant interaction between maternal age and current smoking status among men (p_interaction_ = 0.023) but not in women. Synergistic effects were not observed for season of birth or younger siblings.

**Table 4:** Synergistic effects of early life factors and participants’ current smoking status with regard to lung function decline.

		Adjusted difference in FEV1 decline†			
Early life exposure		in ml ‡	CI 95%		p-value
**maternal age >31 yrs.**	**participant smoking**				
No	No	Ref.			
Yes	No	-0.44	-1.92	1.04	0.559
No	Yes	-1.21	-2.87	0.44	0.150
Yes	Yes	-4.20	-6.44	-1.97	0.000
**maternal smoking**	**participant smoking**				
No	No	Ref.			
Yes	No	-1.75	-3.55	0.04	0.056
No	Yes	-1.82	-3.38	-0.25	0.023
Yes	Yes	-4.44	-7.04	-1.85	0.001

^†^ Change in FEV_1_ (ml) by follow up year—a negative coefficient implies more rapid FEV_1_ decline and a positive coefficient implies less rapid decline.

^‡^ Estimates from mixed-effects linear regression, mutually adjusted for all other early life factors and for sex, mid age, mid age square, mid BMI, change in BMI (between survey 1 and 2), height, age at highest education, pack-years, European region (random effect).

CI = Confidence Interval

**Fig 1:**
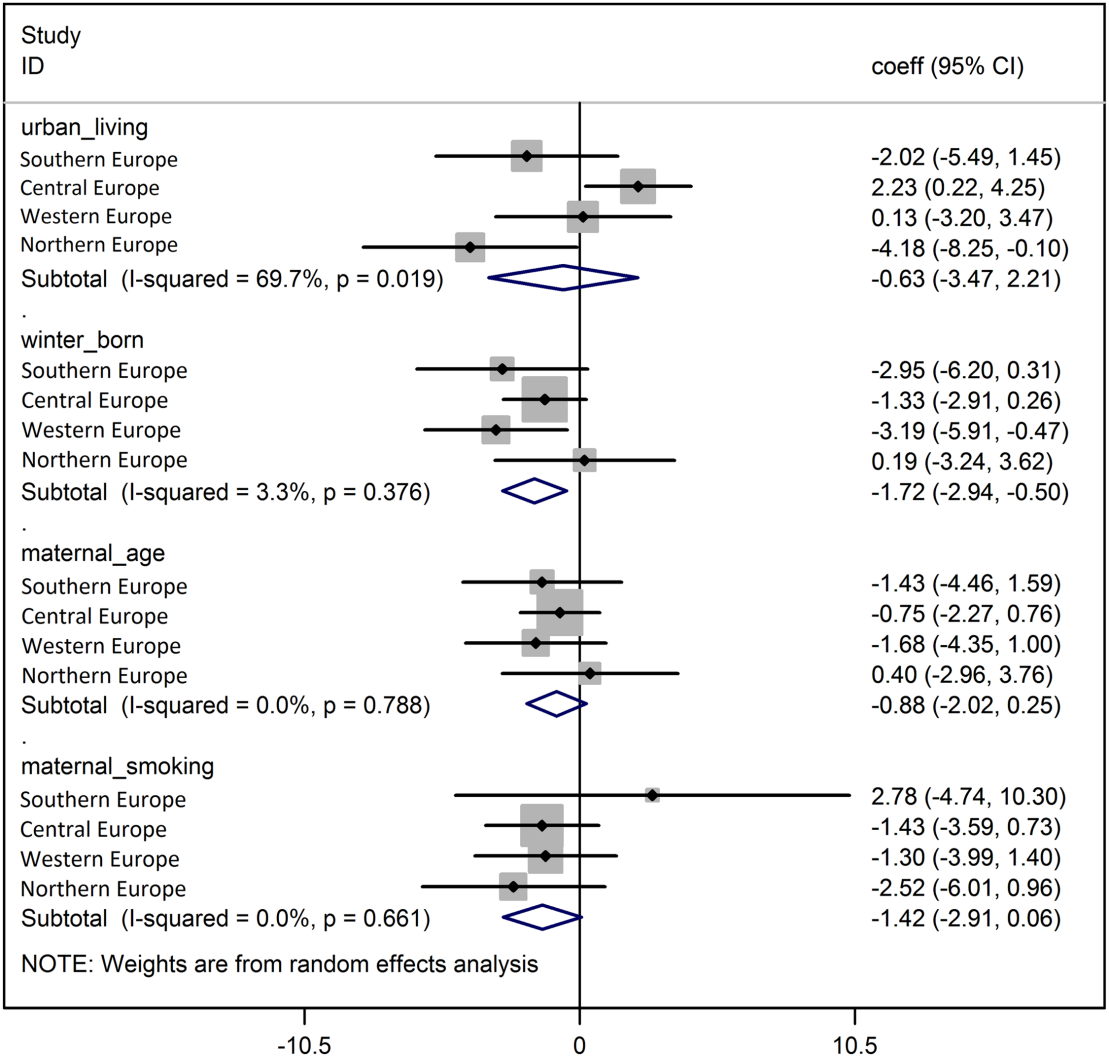
Meta-analyses across European regions: selected early life factors and FEV_1_ decline. ΔFEV1/yr. corresponds to change in FEV1 (ml) per year of follow-up—a negative coefficient implies more rapid FEV1 decline and a positive coefficient implies less rapid decline Meta-Analyses by European region, mutually adjusted for all other early life factors investigated and for sex, mid age, mid age square, mid BMI, change in BMI (between survey 1 and 2), height, lifetime pack years smoked, age at highest education, study area (random effect).

Meta-analyses showed that the associations of early life factors with FEV_1_ decline were generally consistent across European regions (adjusted for study centres within each region) (Fig 1, S2 Fig). Only the association with urban living environment showed a significant heterogeneity, with protective effects in Central Europe whereas adverse effects were present in Western and Southern Europe (heterogeneity p-value 0.016, Fig 1).

## Discussion

In two European multi-centre cohort studies, including 12862 persons aged 28–73 at follow-up and relying on comparable methodology, we found that early life factors predicted lung function decline into old age, suggesting that lung ageing is programmed early in life. Our analyses show a substantial impact on lung function decline by early life factors, ranging from adverse effects almost as large as the effects of personal smoking, to “protective” effects of similar magnitude for having older siblings. The early life impact was independent of participant’s lifetime asthma status. A more rapid lung function decline was associated with season of birth, higher maternal age at delivery, maternal smoking and the presence of younger siblings, whereas early day-care attendance, older siblings and childhood pet keeping appeared to be associated with a less rapid decline. One must consider, that these early life factors may reflect underlying mechanisms, [34] having led to or potentiated the observed lung function decline. The main findings were consistent across European regions with the exception of urban living environment. There was some evidence of a differential impact of early life factors by sex, suggesting that early life programming may be related to gender. Accelerated decline related to adverse early life factors was more pronounced among current smokers than never-smokers, possibly pointing to an increased susceptibility to smoking in subjects exposed to higher maternal age and maternal smoking.

This is the first study to investigate early life impact on lung function decline into old age, and to address the hypothesis on interaction with adult insults like smoking. Our findings agree with some of the earlier studies showing association between early life environment, e.g. maternal smoking, and lung function decline in younger adults.[6, 21, 26, 27]. We did not observe a significant effect for respiratory disease on lung function decline, neither did Svanes et al.[21] or Marossy et al.,[25] although early infections have been associated with lung growth and function.[15] In our study, non-differential misclassification by retrospective recall or different severity of childhood disease may explain the null-finding. Such imprecision would most likely attenuate the true association.

Among the novel findings is the association of lung function decline with season of birth. This association was strong and consistent between European regions. Participants born in winter months had a more rapid decline in lung function. Being born in winter has been related to in-utero exposures to viral infections or allergens and to a higher frequency of respiratory infections in the first months of life,[35] both likely major influences on the subsequent establishment of immune response.[36] Low maternal vitamin D levels, more frequent in winter season, have also been discussed as possible factor influencing childhood wheeze and asthma.[37] Season of birth cannot be separated, however, from season of conception and pregnancy. Our finding that season of birth is somehow related to early life programming of lung ageing should fuel further research into underlying mechanisms.

We found a more pronounced lung function decline in participants born to older mothers. Maternal age at delivery reflects biological ageing of the mother as well as sociocultural characteristics. Pregnancy complications [38] and Caesarean section are more common in older mothers, and may be of importance for immunological functions in infants [39] and beyond [40]. A birth cohort effect may also play a role, as participants born between 1930 and 1950 were more often born to older mothers than younger participants (data not shown). Our result conflict with earlier publications,[41, 42] but are in line with a recent study by Caudri et al. observing a differential impact of maternal age depending on the wheeze phenotypes, implying older age to be associated with late onset wheeze. [43] We also observed that maternal smoking is a significant predictor for accelerated lung function decline even in older age. Long-term impact of maternal smoking have previously been shown with regard to asthma [5] and lung function in young to mid-age adults.[21, 26, 27] Inflammatory pathways are believed to be central; however, many open questions remain regarding the underlying mechanisms.[26, 34] In our cohorts lung function decline was more pronounced in smokers who in addition were exposed to either maternal smoking or higher maternal age. This indicates that susceptibility to later adult insults might be programmed early in life. A synergistic effect of parental smoking has been described by Guerra et al. [26] who found a steeper decline of lung function in young adults exposed to both parental and individual smoking. Upton et al. showed that the effect on airflow limitation by 10 cigarettes/day maternal smoking was numerically equivalent to 10 years active smoking. [27] The importance of the early life environment for susceptibility to adult insults has already been discussed by Ramsey et al., who found an additive influence on FEV_1_ decline by parental and individual socio-economic position. Previous findings on synergistic effects support our novel finding on an increased susceptibility to smoking in subjects born to older mothers. Our data further support some heterogeneity by gender: we found a stronger overall impact of maternal age and current smoking in men as compared to women, even after adjustment for pack years. There are several known mechanisms which may explain different susceptibility by sex to life-time exposures, [13, 44, 45] and several studies have shown a higher respiratory vulnerability in men. [5, 46–48] The concept of increased vulnerability at certain time-points in life is also underlying the analyses of both older and younger siblings, representing an early and a later prolonged exposure to a variety of microbes. While the effect of older siblings was non-significant, we found an independent and adverse effect of the presence of younger siblings. The analytic approach and the result is supported by a publication by Svanes et al. that found a U-shaped association between number of siblings and adult asthma,[23] and a recent publication by Grabenhenrich et al. also observing a U-form association between asthma and age at entering day care.[42]

Day care attendance was related to less rapid lung function decline, independent of gender or smoking status, and similar “protective effects” were indicated for childhood pet keeping and having older siblings. This is the first analysis of lung function decline in relation to these early life exposures. Our findings are in agreement with previous research in younger populations and other respiratory health outcomes, in particular allergic diseases.[49] The data imply that these factors, generally discussed in view of the hygiene hypothesis,[50] can induce long-term modifications of the immune system.

While a study within ECRHS showed that some childhood exposures are reported with high consistency, irrespective of respiratory health status,[51] residual confounding due to recall bias cannot be excluded. All factors except maternal age were recorded at baseline, prior to measurement of lung function at baseline and at follow-up. The retrospective reporting of these factors might have led to imprecise recall, which is likely to have attenuated true associations. It is, however, unlikely that recall would be differential with regard to the outcome of the present analysis—the difference between two objectively measured lung function values. It is also unlikely that recall bias should be uniform across different cohorts and European countries.

The consistent results across European regions suggest that the associations may be due to homogeneous biological mechanisms rather than heterogeneous socio-cultural differences. Heterogeneity between regions was only found for the impact of urban living environment. We observed a significant protective effect on lung function decline by urbanity in Central Europe, while the opposite was found in Western and Southern Europe. Urbanity can be considered a proxy for opposite health determinants, e.g. higher pollutant exposure versus better access to health care. Our data did not allow investigating the latent construct urbanity any further.

With respect to parental smoking, we could not assess how much parents have smoked and for how long. This limitation possibly reduced the power of our study, since one would assume that the synergy would increase with higher doses of exposure in childhood.[27] Residual confounding is also possible due to a lack of information on further potentially influencing factors e.g. nutrition, air pollution exposure in childhood or birth characteristics. Individuals with particularly poor early life development could possibly be more or less likely to participate in the cohort, thus, the prevalence of early life factors might not reflect the true population prevalence. In view of our study hypothesis, this bias would limit the generalizability of our study, but not produce spurious associations. As some study participants were younger than 25 years at the first survey, late lung growth may have confounded the analyses. However, excluding these subjects resulted in consistent associations. The analytical strategy, including all factors in mutually adjusted analyses, may have introduced over-adjustment, potentially reducing the power to identify less strong direct associations.

Investigating early life factors and potential interactions demands large study populations, which was achieved by combining the cohorts SAPALDIA and ECRHS. We had the power to study even potentially small effects of early life factors and to identify sub-groups at risk, given the inclusion of persons in the 7^th^ decade with the advantage of rich information about declining lung function in this aging cohort. This multinational study population constitutes both strengths and limitations. On one hand, our homogenous results across the European regions underline the importance of early life environment in Europe. On the other hand regional differences in the effects of childhood factors may attenuate the overall effects when adjusted for regions and regional specific risks may not have been picked up. The results cannot be generalized to countries where the investigated childhood factors, such as day care, must be considered proxies of considerably different exposures.

In conclusion, our results showed that lung function decline into old age were predicted by early life factors. This novel finding supports the hypothesis that lung ageing, which is captured by lung function decline, is programmed early in life. We further found that lung function decline was more rapid in those with early life disadvantages who subsequently smoked, suggesting that altered individual susceptibility to adult insults could be one possible pathway for persisting effects of early life factors decades later. Interestingly, lung function was found to decline more rapidly in people born during the winter season, consistently across European regions; this effect merits further investigation. From a public health point of view, early life impact on lung function decline and on susceptibility to adult exposures offers possibilities for intervention, and from a clinical point of view, these findings identify a population at risk for accelerated lung function decline.

## Supporting Information

S1 Fig European map and regional distribution of SAPALDIA and ECRHS study centers. (PDF)

S2 Fig Meta-analyses across European regions: Association of early life factors and lung function decline. (PDF)

S1 Table Impact of single early life factors on lung function decline. (PDF)

S2 Table Impact of early life factors on lung function decline, stratified by sex. (PDF)

S3 Table Impact of early life factors on lung function decline, stratified by smoking status. (PDF)

S4 Table Sensitivity analyses. (PDF)

S5 Table Sensitivity analyses: Association between lung function decline and early life factors adjusting for childhood and adult asthma. (PDF)

S6 Table Definitions of early life factors as used in SAPALDIA and ECRHS questionnaires. (PDF)

S1 Text Funding and ethical committees. (DOCX)

S2 Text Cohort funding information. (DOC)
