# Supplementary material for: Early Life Origins of Lung Ageing: Early Life Exposures and Lung Function Decline in Adulthood in Two European Cohorts Aged 28-73 Years
Source: PLoS One. 2016 Jan 26;11(1):e0145127. doi: 10.1371/journal.pone.0145127 (PMC4728209; doi:10.1371/journal.pone.0145127)

Early life origins of lung ageing
Julia Dratva et al.

S- Figure 1: European map and regional distribution of SAPALDIA and ECRHS study centers

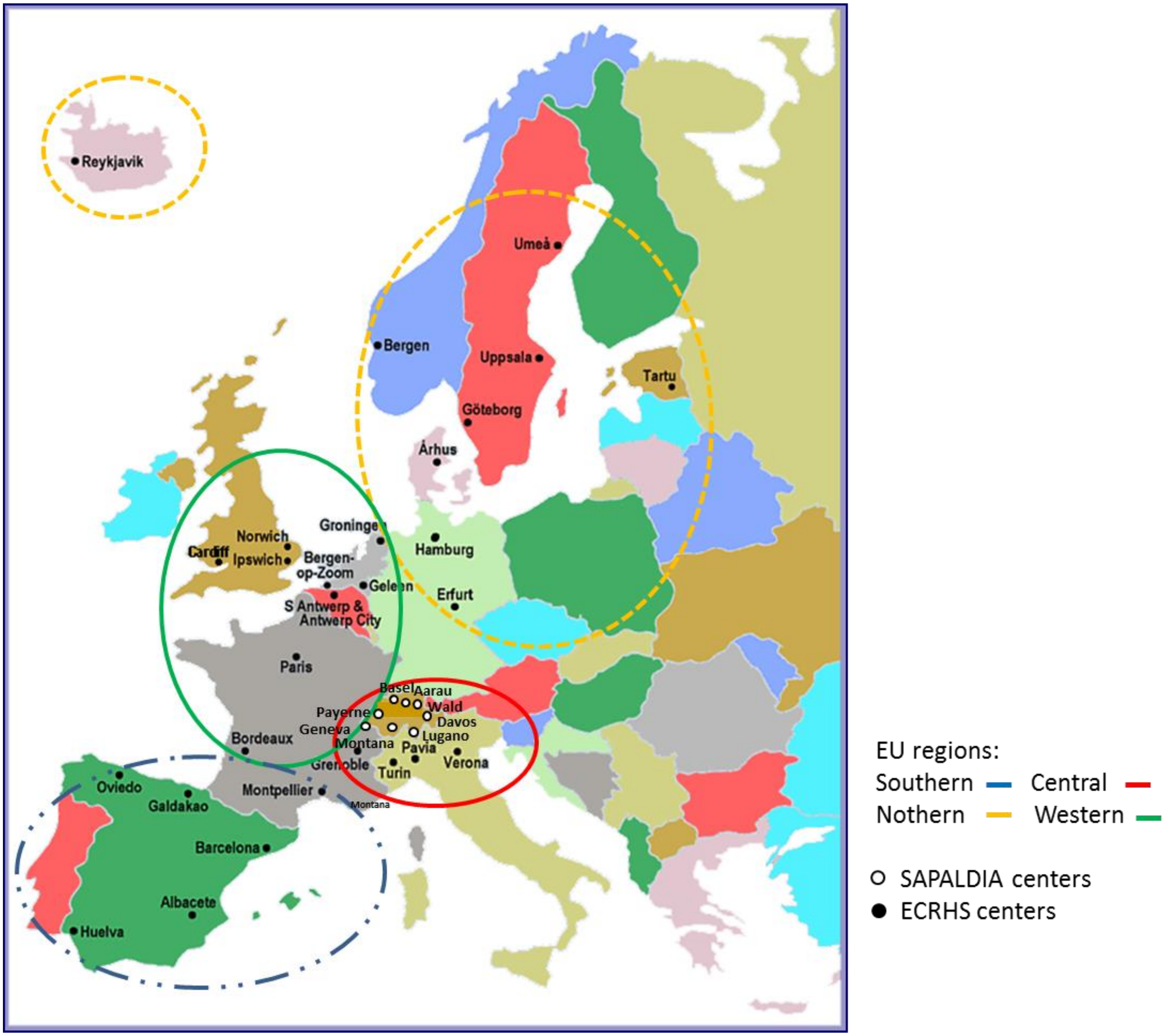

Supplement: S1 Fig — (PDF) [file pone.0145127.s001.pdf]
