# Supplementary material for: Early Life Origins of Lung Ageing: Early Life Exposures and Lung Function Decline in Adulthood in Two European Cohorts Aged 28-73 Years
Source: PLoS One. 2016 Jan 26;11(1):e0145127. doi: 10.1371/journal.pone.0145127 (PMC4728209; doi:10.1371/journal.pone.0145127)
Supplement: S2 Fig — (PDF) [file pone.0145127.s002.pdf]

Early life origins of lung ageing

Julia Dratva et al.

S-Figure 2: Meta-analyses across European regions: Association of early life factors and lung function decline $^{\alpha}$

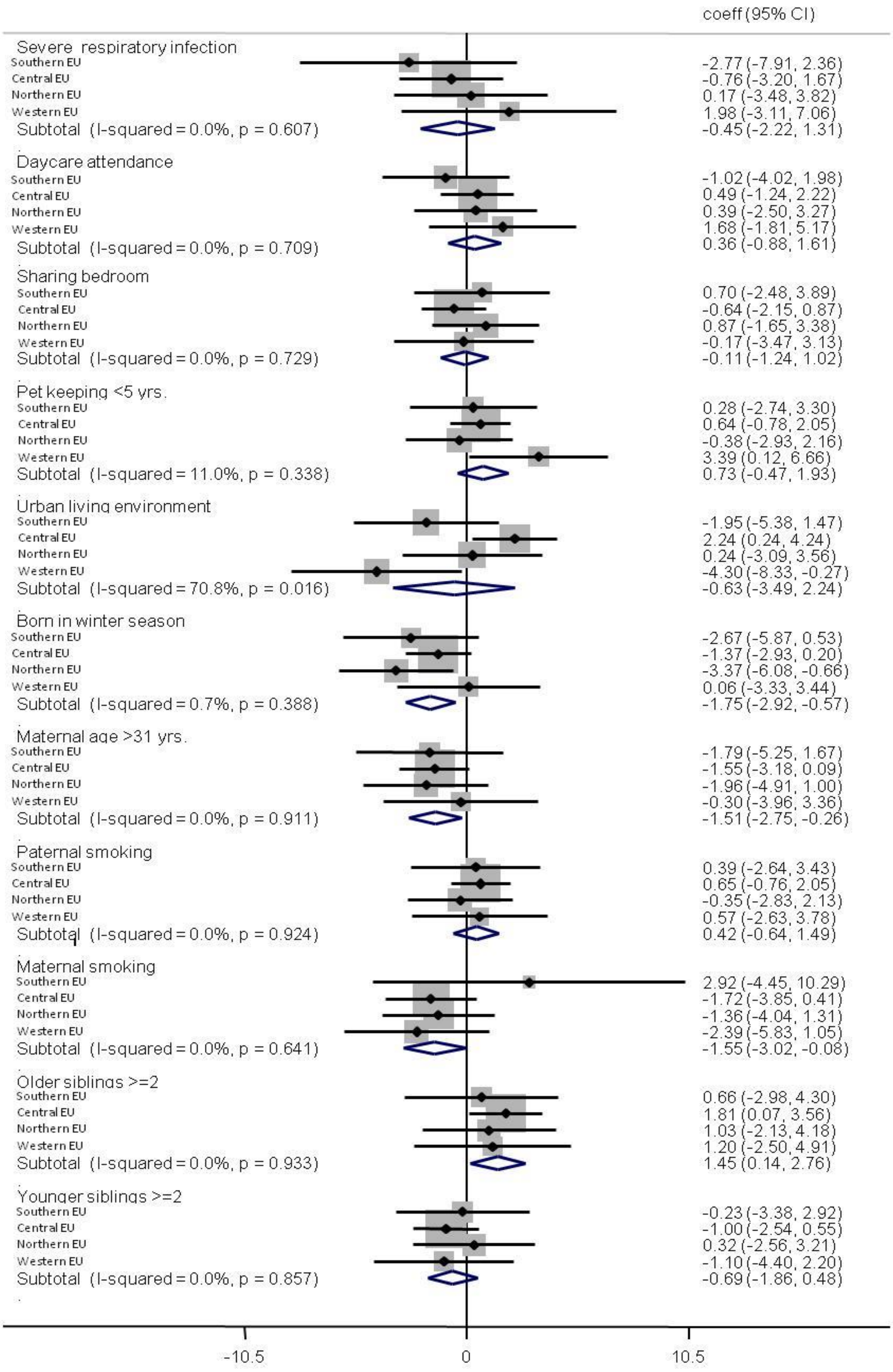

ΔFEV_1/yr = adjusted change in FEV1 (ml) per year of follow-up

α mutually adjusted for all other early life factors investigated and for sex, mid age, mid age square, mid BMI, change in BMI (between survey 1 and 2), height, pack years smoked, age at highest education, study area (random effect)
