# Supplementary material for: Early Life Origins of Lung Ageing: Early Life Exposures and Lung Function Decline in Adulthood in Two European Cohorts Aged 28-73 Years
Source: PLoS One. 2016 Jan 26;11(1):e0145127. doi: 10.1371/journal.pone.0145127 (PMC4728209; doi:10.1371/journal.pone.0145127)
Supplement: S1 Table — (PDF) [file pone.0145127.s003.pdf]

Early life origins of lung ageing
Julia Dratva et al.

S-Table 1: Impact of single early life factors on lung function decline ‡

Early life factors	ΔFEV_1/yr. †	95% CI	p-value
Season of birth: winter	-2.00	-3.25, -0.75	<0.001
Maternal age (>31 yrs.)	-1.12	-2.31; 0.06	<0.001
Maternal smoking	-1.42	-2.87; 0.04	0.02
Paternal smoking	0.38	-0.74; 1.50	0.32
Severe respiratory infection	-0.36	-2.22; 1.50	0.57
Urban living environment	0.61	-0.85; 2.07	0.41
Daycare attendance	3.92	2.72; 5.12	<0.001
Sharing bedroom	-0.46	-1.55; 0.06	0.28
Family pet (< 5yrs.)	0.62	-0.50; 1.73	0.12
Older siblings ≥2	0.24	-0.94; 1.42	0.13
Younger siblings <2	-2.13	-3.32; -0.95	<0.001

† ΔFEV_1/yr. corresponds to change in FEV_1 (ml) by follow up year – a negative coefficient implies more rapid FEV_1 decline and a positive coefficient implies less rapid decline.
‡ adjusted for sex, mid age, mid age square, mid BMI, change in BMI (between survey 1 and 2), height, pack years smoked, age at highest education, European region (random effect)
CI = Confidence Interval
