# Supplementary material for: Early Life Origins of Lung Ageing: Early Life Exposures and Lung Function Decline in Adulthood in Two European Cohorts Aged 28-73 Years
Source: PLoS One. 2016 Jan 26;11(1):e0145127. doi: 10.1371/journal.pone.0145127 (PMC4728209; doi:10.1371/journal.pone.0145127)
Supplement: S2 Table — (PDF) [file pone.0145127.s004.pdf]

Early life origins of lung ageing
Julia Dratva et al.

S-Table 2: Impact of early life factors on lung function decline, stratified by sex‡

	Women N= 6590				Men N= 6163			
Early life factors	ΔFEV/yr. †	95% CI		p-value	ΔFEV/yr. †	95% CI		p-value
Season of birth: winter	-1.59	-3.11	-0.08	0.04	-2.35	-4.38	-0.39	0.02
Maternal age (>31 yrs.)	-2.60	-4.19	-1.00	0.00	-1.04	-3.18	1.10	0.34
Maternal smoking	-0.57	-2.34	1.20	0.53	-2.23	-5.11	-0.33	0.03
Paternal smoking	-0.19	-1.56	1.17	0.78	1.11	-0.78	2.84	0.27
Severe respiratory infection	0.12	-2.03	2.26	0.92	-0.74	-4.33	1.96	0.46
Urban living environment	0.61	-1.14	2.37	0.50	0.56	-1.69	3.05	0.58
Daycare attendance	4.48	3.02	5.95	0.00	3.49	1.50	5.35	0.00
Sharing bedroom	-1.00	-2.39	0.40	0.16	0.06	-1.70	1.81	0.95
Family pet (<5 yrs.)	0.95	-0.41	2.30	0.17	0.83	-1.04	2.55	0.41
Older siblings ≥2	1.85	-0.01	3.71	0.05	-0.70	-3.14	1.50	0.49
Younger siblings <2	-1.91	-3.39	-0.43	0.01	-3.05	-4.91	-1.06	0.00

† ΔFEV_1/yr. corresponds to change in FEV_1 (ml) by follow up year – a negative coefficient implies more rapid FEV_1 decline and a positive coefficient implies less rapid decline.
‡ adjusted for mid age, mid age square, mid BMI, change in BMI (between survey 1 and 2), height, pack years smoked, age at highest education, European region (random effect)
CI = Confidence Interval
