# Supplementary material for: Early Life Origins of Lung Ageing: Early Life Exposures and Lung Function Decline in Adulthood in Two European Cohorts Aged 28-73 Years
Source: PLoS One. 2016 Jan 26;11(1):e0145127. doi: 10.1371/journal.pone.0145127 (PMC4728209; doi:10.1371/journal.pone.0145127)
Supplement: S3 Table — (PDF) [file pone.0145127.s005.pdf]

**Early life origins of lung ageing**
Julia Dratva et al.

S-Table 3: Impact of early life factors on lung function decline, stratified by smoking status‡

| | Never smokers N=5323 | | | | Current smokers N=3527 | | | |
|---|---|---|---|---|---|---|---|---|
| Early life factors | $\Delta FEV_1$/yr. † | p-value | 95% CI | | $\Delta FEV_1$/yr. † | p-value | 95% CI | |
| Season of birth: winter | -2.46 | 0.011 | -4.35 | -0.57 | -1.71 | 0.183 | -4.23 | 0.81 |
| Maternal age (>31 yrs.) | -1.34 | 0.196 | -3.37 | 0.69 | -3.23 | 0.018 | -5.90 | -0.56 |
| Maternal smoking | -1.44 | 0.217 | -3.73 | 0.85 | -3.77 | 0.011 | -6.67 | -0.87 |
| Paternal smoking | 0.81 | 0.339 | -0.86 | 2.48 | -0.50 | 0.680 | -2.86 | 1.86 |
| Severe respiratory infection | 0.44 | 0.761 | -2.38 | 3.26 | -1.63 | 0.385 | -5.31 | 2.05 |
| Urban living environment | 0.20 | 0.860 | -1.97 | 2.37 | 0.74 | 0.631 | -2.29 | 3.77 |
| Daycare attendance | 3.38 | 0.000 | 1.56 | 5.19 | 4.29 | 0.000 | 1.93 | 6.64 |
| Sharing bedroom | 0.26 | 0.771 | -1.50 | 2.02 | -0.50 | 0.676 | -2.82 | 1.83 |
| Family pet (<5 yrs.) | 1.81 | 0.037 | 0.11 | 3.51 | 1.05 | 0.367 | -1.24 | 3.35 |
| Older siblings ≥2 | 1.26 | 0.310 | -1.17 | 3.69 | 1.09 | 0.482 | -1.95 | 4.14 |
| Younger siblings <2 | -2.16 | 0.023 | -4.02 | -0.30 | -3.05 | 0.017 | -5.55 | -0.54 |

† $\Delta FEV_1$/yr. corresponds to change in $FEV_1$ (ml) by follow up year – a negative coefficient implies more rapid $FEV_1$ decline and a positive coefficient implies less rapid decline.
‡ mutually adjusted for all other early life factors investigated and sex, mid age, mid age square, mid BMI, change in BMI (between survey 1 and 2), height, pack years smoked (in smokers), age at highest education, European region (random effect)
CI = Confidence Interval
