# Supplementary material for: Early Life Origins of Lung Ageing: Early Life Exposures and Lung Function Decline in Adulthood in Two European Cohorts Aged 28-73 Years
Source: PLoS One. 2016 Jan 26;11(1):e0145127. doi: 10.1371/journal.pone.0145127 (PMC4728209; doi:10.1371/journal.pone.0145127)
Supplement: S4 Table — (PDF) [file pone.0145127.s006.pdf]

**Early life origins of lung ageing**
Julia Dratva et al.

S-Table 4: Sensitivity analyses

Association between lung function decline and early life factors‡, excluding
a) participants with reported childhood and b) adult asthma, c) COPD or d) >25 yrs. of age at first survey

| | Subjects reporting | | | | | | | |
|---|---|---|---|---|---|---|---|---|
| | a) childhood asthma excluded N=11717 | | | | b) adult asthma excluded N= 10933 | | | |
| Early life factors | ΔFEV/yr. † | 95% CI | | p-value | ΔFEV/yr. † | 95% CI | | p-value |
| Season of birth: winter | -2.11 | -3.38 | -0.84 | 0.00 | -1.88 | -3.17 | -0.59 | 0.00 |
| Maternal age † (>31yrs.) | -1.89 | -3.21 | -0.56 | 0.01 | -1.95 | -3.29 | -0.61 | 0.00 |
| Maternal smoking | -1.45 | -2.97 | 0.07 | 0.06 | -1.52 | -3.08 | 0.04 | 0.06 |
| Paternal smoking | 0.71 | -0.45 | 1.87 | 0.23 | 0.67 | -0.50 | 1.84 | 0.26 |
| Severe respiratory infection | -0.37 | -2.35 | 1.62 | 0.72 | -0.19 | -2.19 | 1.81 | 0.85 |
| Urban living environment | 0.14 | -1.37 | 1.65 | 0.86 | 0.21 | -1.32 | 1.74 | 0.79 |
| Daycare attendance | 4.39 | 3.16 | 5.62 | 0.00 | 4.49 | 3.24 | 5.74 | 0.00 |
| Sharing bedroom | -0.57 | -1.77 | 0.62 | 0.35 | 0.10 | -1.11 | 1.31 | 0.87 |
| Family pet (<5 yrs.) | 1.02 | -0.13 | 2.17 | 0.08 | 0.74 | -0.43 | 1.91 | 0.21 |
| Older siblings ≥2 | 0.48 | -1.08 | 2.04 | 0.54 | -0.32 | -1.92 | 1.29 | 0.70 |
| Younger siblings <2 | -2.23 | -3.47 | -0.98 | 0.00 | -2.28 | -3.54 | -1.02 | 0.00 |

| | c) COPD § excluded N=11638 | | | | d) Subjects >25 yrs. old at first survey N= 10770 | | | |
|---|---|---|---|---|---|---|---|---|
| Early life factors | ΔFEV/yr.† | 95% CI | | p-value | ΔFEV/yr.† | 95% CI | | p-value |
| Season of birth: winter | -2.00 | -3.25 | -0.74 | 0.00 | -1.86 | -3.19 | -0.53 | 0.01 |
| Maternal age † (>31yrs.) | -1.90 | -3.20 | -0.60 | 0.00 | -1.35 | -2.72 | 0.03 | 0.06 |
| Maternal smoking | -2.07 | -3.56 | -0.58 | 0.01 | -2.52 | -4.13 | -0.92 | 0.00 |
| Paternal smoking | 0.32 | -0.81 | 1.46 | 0.58 | 0.50 | -0.71 | 1.72 | 0.42 |
| Severe respiratory infection | 0.58 | -1.30 | 2.46 | 0.55 | -0.96 | -2.92 | 1.01 | 0.34 |
| Urban living environment | 0.01 | -1.47 | 1.48 | 0.99 | -0.07 | -1.66 | 1.52 | 0.93 |
| Daycare attendance | 4.43 | 3.22 | 5.63 | 0.00 | 3.78 | 2.48 | 5.07 | 0.00 |
| Sharing bedroom | -0.63 | -1.80 | 0.54 | 0.29 | -1.15 | -2.40 | 0.09 | 0.07 |
| Family pet (< 5 yrs.) | 0.91 | -0.22 | 2.04 | 0.11 | 0.93 | -0.27 | 2.13 | 0.13 |
| Older siblings ≥2 | 0.41 | -1.13 | 1.95 | 0.60 | 1.19 | -0.43 | 2.82 | 0.15 |
| Younger siblings <2 | -2.37 | -3.59 | -1.14 | 0.00 | -2.24 | -3.53 | -0.95 | 0.00 |

† $\Delta FEV_1$/yr. corresponds to change in $FEV_1$ (ml) by follow up year – a negative coefficient implies more rapid $FEV_1$ decline and a positive coefficient implies less rapid decline.
