# Supplementary material for: Early Life Origins of Lung Ageing: Early Life Exposures and Lung Function Decline in Adulthood in Two European Cohorts Aged 28-73 Years
Source: PLoS One. 2016 Jan 26;11(1):e0145127. doi: 10.1371/journal.pone.0145127 (PMC4728209; doi:10.1371/journal.pone.0145127)
Supplement: S5 Table — (PDF) [file pone.0145127.s007.pdf]

Early life origins of lung ageing
Julia Dratva et al.

S-Table 5: Sensitivity analyses: Association between lung function decline and early life factors adjusting for childhood and adult asthma‡§

	adjusted for childhood asthma N=12192				adjusted for adult asthma N= 11648			
Early life factors	ΔFEV/yr. †	95% CI		p-value	ΔFEV/yr. †	95% CI		p-value
Season of birth: winter	-2.14	-3.41	-0.87	0.00	-1.96	-3.23	-0.69	0.00
Maternal age † (>31yrs.)	-1.83	-3.15	-0.50	0.01	-1.85	-3.17	-0.53	0.01
Maternal smoking	-1.77	-3.28	-0.26	0.02	-1.49	-3.01	0.03	0.05
Paternal smoking	0.45	-0.71	1.61	0.44	0.69	-0.47	1.84	0.24
Severe respiratory infection	-0.38	-2.29	1.53	0.70	-0.33	-2.27	1.62	0.74
Urban living environment	0.35	-1.15	1.84	0.65	0.31	-1.19	1.81	0.69
Daycare attendance	4.17	2.94	5.39	0.00	4.44	3.22	5.66	0.00
Sharing bedroom	-0.48	-1.67	0.71	0.43	-0.37	-1.56	0.81	0.54
Family pet (< 5 yrs.)	1.18	0.03	2.33	0.04	1.08	-0.07	2.23	0.07
Older siblings ≥2	0.56	-1.00	2.12	0.48	-0.02	-1.58	1.54	0.98
Younger siblings <2	-2.50	-3.74	-1.25	0.00	-2.25	-3.48	-1.01	0.00

† ΔFEV_1/yr. corresponds to change in FEV_1 (ml) by follow up year – a negative coefficient implies more rapid FEV_1 decline and a positive coefficient implies less rapid decline.
‡ mutually adjusted for all other early life factors investigated and sex, mid age, mid age square, mid BMI, change in BMI (between survey 1 and 2), height, pack years smoked, age at highest education, European region (random effect)
§ child asthma adjusted: N=11981, adult asthma adjusted N=11449
