# Supplementary material for: Early Life Origins of Lung Ageing: Early Life Exposures and Lung Function Decline in Adulthood in Two European Cohorts Aged 28-73 Years
Source: PLoS One. 2016 Jan 26;11(1):e0145127. doi: 10.1371/journal.pone.0145127 (PMC4728209; doi:10.1371/journal.pone.0145127)
Supplement: S6 Table — (PDF) [file pone.0145127.s008.pdf]

Early life origins of lung ageing

Julia Dratva et al.

S-Table 6: Definitions of early life factors as used in SAPALDIA and ECRHS questionnaires

Early life exposure factor	Question assessing exposure	Coding
severe respiratory infections	Were you hospitalized before the age of two years for lung disease?	yes/no
sharing bedroom	How many other children regularly slept in your bedroom before you were five years old?	number
daycare attendance	Did you go to a school, play-school or nursery with older children before the age of five years?	yes/no
family pet <5yrs (combining question on pet keeping in first year of life and 1-4 years of age)	Was there a cat/dog/bird in your home? During your first year of life?	yes/no to each animal separately
	When you were aged 1 to 4 years?	yes/no to each animal separately
	When you were aged 5-15 years?	yes/no to each animal separately
paternal smoking	Did your father ever smoke regularly during your childhood?	yes/no
maternal smoking	Did your mother ever smoke regularly during your childhood or before you were born?	yes/no
maternal age (dichotomized for analyses: maternal age <=31/>31 yrs.)	How old was your mother when you were born?	age in years
season of birth (dichotomized for analyses: spring, summer, autumn/winter)		based on month of birth
living environment (dichotomized for analyses: non-urban/urban)	What term best describes the place you lived most of the time when you were under the age of five years?	Farm/ village in rural area/small town/ suburb in large town/ town centre in large town
number of siblings (dichotomized for analyses: <3 sibling/>=3 siblings)	How many brothers/sisters do or did you have?	number
number of older siblings (dichotomized for analyses: <2/>=2 older siblings)	How many older brothers/sisters?	number
