# Supplementary material for: Early Life Origins of Lung Ageing: Early Life Exposures and Lung Function Decline in Adulthood in Two European Cohorts Aged 28-73 Years
Source: PLoS One. 2016 Jan 26;11(1):e0145127. doi: 10.1371/journal.pone.0145127 (PMC4728209; doi:10.1371/journal.pone.0145127)
Supplement: S1 Text — (DOCX) [file pone.0145127.s009.docx]

S1- Text – Funding and ethical committees

**Early Life Origins of Lung Ageing: Early Life Exposures and Lung Function Decline in Adulthood in Two European Cohorts Aged 28-73 Years**

Julia Dratva^1,2^, Elisabeth Zemp^1,2^, Shyamali C Dharmage^3^ , Simone Accordini^4^, Luc Burdet^5^, Thorarinn Gislason^6^, Joachim Heinrich^7^, Christer Janson ^8^, Deborah Jarvis^9^, Roberto de Marco^4^, Dan Norbäck^10^, Marco Pons ^11^, Francisco Gómez Real^12,13^, Jordi Sunyer ^14^, Simona Villani^15^, Nicole Probst-Hensch^1,2^, Cecilie Svanes^16,17^

**Funding (short, more detailed in S2-Text):**

The first author was supported by a Marie Heim-Vögtlin grant from the Swiss National Science Foundation (grant # PMPDP3_129021/1; # PMPDP3_141671/1), the Lung league Beider Basel, Lung league Graubünden, the Stiftung ehemals Bündner Heilstätten and the COST action BM1201. Research support received by the SAPALDIA and ECRHS cohorts can be found in the online supplement (funding-supplement). Prof. Cecilia Svanes and Prof. Thorarinn Gislason have, as members of the COST BMI 1201, received financial support from COST BMI 1201.

**Ethical Committees**

SAPALDIA and ECRHS were evaluated and granted permission by the following ethical committees

SAPALDIA: Ethikkommission Kantonspital Aarau ; Ethikkommission Beider Basel, COMITATO ETICO CANTONALE; Kantonale Ethikkommission Graubünden; Comité d’Ethique de département du médicine – Hôpital Universtiaire Genève; Comité d’Ethique de la recherche clinique – Faculté de médicine Lausanne; Commission d‘ éthique de la Société médicale du Valais – Sion; Ethisches Komitee des Departments für Innere Medizin, Universität Zürich.

ECRHS: Sweden: Regional ethical committee of Uppsala (N° 2010/068); Germany:Ethikkommission der Bayerischen Ärztekammer (Votum Nr. 10015); Italy: Ethiclal boards of Turin: Azienda Sanitaria Locale TO-2; Pavia: ‘‘Istituto Ricovero e Cura a Carattere Scientifico’’, Policlinico San Matteo; "Ethical Board of Department of Internal Medicine - Pavia University". Verona: ‘‘Istituti Ospedalieri di Verona’’; Norway: Regional Ethics Committee of Western Norway; Iceland: [The National Bioethics Committee](http://www.vsn.is/en); Spain:

Comité Ético de Investigación Clínica del Instituto Municipal de Asistencia Sanitaria, Barcelona, Comité Ético de Investigación del Hospital de Galdakao, Albacete: Comité de Ética e Investigación de Complejo Hospitalario de Albacete, Oviedo: Comité Ético de Investigación Clínica Regional, Hospital Universitario Central de Asturias, Oviedo,

Huelva: Comisión de Investigación del Hospital Juan Ramón Jiménez de Huelva; Estland: Research Ethics Committee of the University of Tartu in Estonia; France: French Institutional Ethics Committee; Great-Britain: [Imperial College Research Ethics Committee](http://www.imperial.ac.uk/research-and-innovation/support-for-staff/research-ethics-committee/).

Data availability:

At the time of SAPALDI 1 and 2 and ECRHS I and II providing data publically was not considered and participants were not asked for consent to provide individual data to the public. The data are available to scientists upon request to the study primary study investigators (*www.ecrhs.org/ ;* [*www.sapaldia.ch*](http://www.sapaldia.ch)*)* or via contact with the first and last authors of the manuscript.
