# Supplementary material for: Early Life Origins of Lung Ageing: Early Life Exposures and Lung Function Decline in Adulthood in Two European Cohorts Aged 28-73 Years
Source: PLoS One. 2016 Jan 26;11(1):e0145127. doi: 10.1371/journal.pone.0145127 (PMC4728209; doi:10.1371/journal.pone.0145127)
Supplement: S2 Text — (DOC) [file pone.0145127.s010.doc]

# S2-Text Cohort funding information

**PLOS One**

**Early life origins of lung ageing: Early life exposures and lung function decline in adulthood in two European cohorts aged 28-73 years**

Julia Dratva^1,2^, Elisabeth Zemp^1,2^, Shyamali C Dharmage^3^ , Simone Accordini^4^, Luc Burdet^5^, Thorarinn Gislason^6^, Joachim Heinrich^7^, Christer Janson ^8^, Deborah Jarvis^9^, Roberto de Marco^4^, Dan Norbäck^10^, Marco Pons ^11^, Francisco Gómez Real^12,13^, Jordi Sunyer ^14^, Simona Villani^15^, Nicole Probst-Hensch^1,2^, Cecilie Svanes^16,17^

Study organization and research funding

SAPALDIA

**Current SAPALDIA Team**

**Study directorate**: T Rochat (p), NM Probst Hensch (e/g), N Künzli (e/exp), C Schindler (s), JM Gaspoz (c)

**Scientific team**: JC Barthélémy (c), W Berger (g), R Bettschart (p), A Bircher (a), O Brändli (p), C Brombach (n), M Brutsche (p), L Burdet (p), M Frey (p), U Frey (pd), MW Gerbase (p), D Gold (e/c/p), E de Groot (c), W Karrer (p), R Keller (p), B Martin (pa), D Miedinger (o), U Neu (exp), L Nicod (p), M Pons (p), F Roche (c), T Rothe (p), E Russi (p), P Schmid-Grendelmeyer (a), A Schmidt-Trucksäss (pa), A Turk (p), J Schwartz (e), D. Stolz (p), P Straehl (exp), JM Tschopp (p), A von Eckardstein (cc), E Zemp Stutz (e).

**Scientific team at coordinating centers**: M Adam (e/g), C Autenrieth (pa), PO Bridevaux (p), D Carballo (c), E Corradi (e), I Curjuric (e), J Dratva (e), A Di Pasquale (s), E Dupuis Lozeron (s), E Fischer (e), M Germond (s), L Grize (s), D Keidel (s), S Kriemler (pa), A Kumar (g), M Imboden (g), N Maire (s), A Mehta (e), H Phuleria (exp), E Schaffner (s), GA Thun (g) A Ineichen (exp), M Ragettli (e), M Ritter (exp), T Schikowski (e), M Tarantino (s), M Tsai (e)

(a) allergology, (c) cardiology, (cc) clinical chemistry, (e) epidemiology, (exp) exposure, (g) genetic and molecular biology, (m) meteorology, (n) nutrition, (o) occupational health, (p) pneumology, (pa) physical activity, (pd) pediatrics, (s) statistics

**Research support**:
The Swiss National Science Foundation (grants no 33CSCO-134276/1, 33CSCO-108796, 3247BO-104283, 3247BO-104288, 3247BO-104284, 3247-065896, 3100-059302, 3200-052720, 3200-042532, 4026-028099, PMPDP3_129021/1, PMPDP3_141671/1), the Federal Office for Forest, Environment and Landscape, the Federal Office of Public Health, the Federal Office of Roads and Transport, the canton's government of Aargau, Basel-Stadt, Basel-Land, Geneva, Luzern, Ticino, Valais, and Zürich, the Swiss Lung League, the canton's Lung League of Basel Stadt/ Basel Landschaft, Geneva, Ticino, Valais, Graubünden and Zurich, Stiftung ehemals Bündner Heilstätten, SUVA, Freiwillige Akademische Gesellschaft, UBS Wealth Foundation, Talecris Biotherapeutics GmbH, Abbott Diagnostics, European Commission 018996 (GABRIEL), Wellcome Trust WT 084703MA.

**Acknowledgements**
The study could not have been done without the help of the study participants, technical and administrative support and the medical teams and field workers at the local study sites.
Local fieldworkers : Aarau: S Brun, G Giger, M Sperisen, M Stahel, Basel: C Bürli, C Dahler, N Oertli, I Harreh, F Karrer, G Novicic, N Wyttenbacher, Davos: A Saner, P Senn, R Winzeler, Geneva: F Bonfils, B Blicharz, C Landolt, J Rochat, Lugano: S Boccia, E Gehrig, MT Mandia, G Solari, B Viscardi, Montana: AP Bieri, C Darioly, M Maire, Payerne: F Ding, P Danieli A Vonnez, Wald: D Bodmer, E Hochstrasser, R Kunz, C Meier, J Rakic, U Schafroth, A Walder.

**Administrative staff**: C Gabriel, R Gutknecht.

ECRHS

List of investigators in the Early life Working Group in ECRHS

Chair: Cecilie Svanes. Members: Shyamali Dharmage, Melbourne, Australia; Thorarinn Gislason, Reykjavik; Maria Gunnbjørnsdottir, Uppsala; Joachim Heinrich, Neuherberg; Deborah Jarvis, London; Benedicte Leynaert, Paris; Dora Ludvigsdottir, Reykjavik; Roberto de Marco, Verona; Francoise Neukirch, Paris; Chantal Raherison, Paris; Jordi Sunyer, Barcelona; Simona Villani, Pavia, Matthias Wjst, Neuherberg, Julia Dratva, Basel

# List of Principal Investigators and Senior Scientific Teams in ECRHS

Belgium: South Antwerp & Antwerp City (P Vermeire, J Weyler, M Van Sprundel, V Nelen). Estonia: Tartu (R Jogi, A Soon). France: Paris (F Neukirch, B Leynaert, R Liard, M Zureik), Grenoble (I Pin, J Ferran-Quentin). Germany: Erfurt (J Heinrich, M Wjst, C Frye, I Meyer). Iceland: Reykjavik (T Gislason, E Bjornsson, D Gislason, T Blondal, KB Jorundsdottir). Italy: Turin (M Bugiani, P Piccioni, E Caria, A Carosso, E Migliore, G Castiglioni), Verona (R de Marco, G Verlato, E Zanolin, S Accordini, A Poli, V Lo Cascio, M Ferrari), Pavia (A Marinoni, S Villani, M Ponzio, F Frigerio, M Comelli, M Grassi, I Cerveri, A Corsico). Netherlands: Groningen & Geleen (J Schouten, M Kerkhof). Norway: Bergen (A Gulsvik, E Omenaas, C Svanes, B Laerum). Spain: Barcelona (JM Antó, J Sunyer, M Kogevinas, JP Zock, X Basagana, A Jaen, F Burgos), Huelva (J Maldonado, A Pereira, JL Sanchez), Albacete (J Martinez-Moratalla Rovira, E Almar), Galdakao (N Muniozguren, I Urritia), Oviedo (F Payo). Sweden: Uppsala (C Janson, G Boman, D Norback, M Gunnbjornsdottir), Goteborg (K Toren, L Lillienberg, AC Olin, B Balder, A Pfeifer-Nilsson, R Sundberg), Umea (E Norrman, M Soderberg, K Franklin, B Lundback, B Forsberg, L Nystrom). Switzerland: Basel (N Künzli, B Dibbert, M Hazenkamp, M Brutsche, U Ackermann-Liebrich). United Kingdom: Norwich (D Jarvis, B Harrison), Ipswich (D Jarvis, R Hall, D Seaton)

# Centres taking part at their own expense: Australia: Melbourne (M Abramson, S Dharmage, J Raven, EH Walters). France: Bordeaux (A Taytard, C Raherison), Montpellier (J Bousquet, P Demoly). Germany: Hamburg (K Richter). USA: Portland (M Osborne, S Buist, W Vollmer, L Johnson).

**Funding**

None of the study sponsors/funders had any role on study design, data collection, data analysis, data interpretation or writing of the report. The corresponding author had full access to all the data in the study and had full responsibility for the decision to submit for publication.

**Financial support for ECRHS I:** The following grants helped to fund the local studies.

**Australia:** Allen and Hanbury's, **Belgium**: Belgian Science Policy Office, National Fund for Scientific Research, **Estonia:** Estonian Science Foundation, grant no 1088, **France:** Ministère de la Santé, Glaxo France, Insitut Pneumologique d'Aquitaine, Contrat de Plan Etat-Région Languedoc-Rousillon, CNMATS, CNMRT (90MR/10, 91AF/6), Ministre delegué de la santé, RNSP, France; GSF, **Germany**: Bundesminister für Forschung und Technologie, **Greece:** The Greek Secretary General of Research and Technology, Fisons, Astra and Boehringer-Ingelheim; **India:** Bombay Hospital Trust, **Italy:** Ministero dell'Università e della Ricerca Scientifica e Tecnologica, CNR, Regione Veneto grant RSF n. 381/05.93, **New Zealand:** Asthma Foundation of New Zealand, Lotteries Grant Board, Health Research Council of New Zealand, **Norway:** Norwegian Research Council project no. 101422/310; **Portugal:** Glaxo Farmacêutica Lda, Sandoz Portugesa, **Spain:** Ministero Sanidad y Consumo FIS (grants #91/0016060/00E-05E and #93/0393), Hospital General de Albacete, Hospital General Juan Ramón Jiménez, Consejeria de Sanidad Principado de Asturias, **Sweden:** The Swedish Medical Research Council, the Swedish Heart Lung Foundation, the Swedish Association against Asthma and Allergy; **Switzerland:** Swiss national Science Foundation grant 4026-28099; **UK:** National Asthma Campaign, British Lung Foundation, Department of Health, South Thames Regional Health Authority**, USA:** United States Department of Health, Education and Welfare Public Health Service (grant #2 S07 RR05521-28).

**Financial Support for ECHRS II: Australia:** National health and Medical Research Council**, Belgium:** Antwerp: Fund for Scientific Research (grant code, G.0402.00), University of Antwerp, Flemish Health Ministry**;** **Estonia: Tartu** Estonian Science Foundation grant no 4350, **France: Bordeaux**: Institut Pneumologique d’Aquitaine; **Grenoble:** Programme Hospitalier de Recherche Clinique—Direction de la Recherche Clinique (DRC) de Grenoble 2000 number 2610, Ministry of Health, Ministere de l’Emploi et de la Solidarite, Direction Generale de la Sante, Centre Hospitalier Universitaire (CHU) de Grenoble, Comite des Maladies Respiratoires de l’Isere; **Montpellier:** Programme Hospitalier de Recherche Clinique—DRC de Grenoble 2000 number 2610, Ministry of Health, Direction de la Recherche Clinique, CHU de Grenoble, Ministere de l’Emploiet de la Solidarite, Direction Generale de la Sante, Aventis ( France), Direction Regionale des Affaires Sanitaires et Sociales Languedoc-Roussillon**; Paris:** Ministere de l’Emploi et de la Solidarite, Direction Generale de la Sante,Union Chimique Belge- Pharma (France),Aventis (France), Glaxo France, Programme Hospitalier de Recherche Clinique—DRC de Grenoble2000 number 2610, Ministry of Health, Direction de la Recherche Clinique, CHU de Grenoble; **Germany: Erfurt** GSF—National Research Centre for Environment and Health, Deutsche Forschungsgemeinschaft (grant code, FR1526/1-1) **Hamburg:** GSF—National Research Centre for Environment and Health, Deutsche Forschungsgemeinschaft (grant code, MA 711/4-1) **Iceland: Reykjavik**, Icelandic Research Council, Icelandic University Hospital Fund; **Italy: Pavia** GlaxoSmithKline Italy, Italian Ministry of University and Scientific and Technological Research (MURST),Local University Funding for Research 1998 and 1999;**Turin**: Azienda Sanitaria Locale 4 Regione Piemonte (Italy), Azienda Ospedaliera Centro Traumatologico Ospedaliero/Centro Traumatologico Ortopedico—Istituto Clinico Ortopedico Regina Maria Adelaide Regione Piemonte **Verona:** Ministero dell’Universita´ e della Ricerca Scientifica (MURST), Glaxo Wellcome spa, **Norway: Bergen:** Norwegian Research Council, Norwegian Asthma and Allergy Association, Glaxo Wellcome AS, Norway Research Fund; **Spain: Albacete**: Fondo de Investigaciones Santarias (grant codes, 97/0035-01,99/0034-01 and 99/0034 02), HospitalUniversitario de Albacete, Consejeria de Sanidad; **Barcelona:** Sociedad Espanola de Neumologı´a y Cirugı´a Toracica, Public Health Service(grant code, R01 HL62633-01), Fondo de Investigaciones Santarias (grant codes, 97/0035-01, 99/0034-01, and 99/0034-02), Consell Interdepartamentalde Recerca i Innovacio´ Tecnolo`gica (grant code, 1999SGR 00241) Instituto de Salud Carlos III; Red deCentros de Epidemiologı´a y Salud Pu´blica, C03/09,Redde Basesmoleculares y fisiolo´gicas de lasEnfermedadesRespiratorias,C03/011and Red de Grupos Infancia y Medio Ambiente G03/176; **Huelva:** Fondo de Investigaciones Santarias (grant codes, 97/0035-01, 99/0034-01, and 99/0034-02); **Galdakao:** Basque Health Department **Oviedo:** Fondo de Investigaciones Santarias (grant codes, 97/0035-01, 99/0034-01, and99/0034-02); **Sweden: Göteborg , Umea, Uppsala**: Swedish Heart Lung Foundation, Swedish Foundation for Health Care Sciences and Allergy Research, Swedish Asthma and Allergy Foundation, Swedish Cancer and Allergy Foundation, Swedish Council for Working Life and Social Research (FAS),**Switzerland: Basel** Swiss National Science Foundation, Swiss Federal Office for Education and Science, Swiss National Accident Insurance Fund; **UK: Ipswich and Norwich**: Asthma UK (formerly known as National Asthma Campaign) **USA: Portland**: American Lung Association of Oregon, Northwest Health Foundation, Collins Foundation, Merck Pharmaceutical.
